# Editorial: Disruption of the Microbiota-Gut-Brain Axis in Functional Dyspepsia and Gastroparesis: Mechanisms and Clinical Implications

**DOI:** 10.3389/fnins.2022.941810

**Published:** 2022-07-05

**Authors:** Lucas Wauters, Hui Li, Nicholas J. Talley

**Affiliations:** ^1^Department of Gastroenterology and Hepatology, University Hospitals Leuven, Leuven, Belgium; ^2^Vagal Afferent Research Group, Adelaide Medical School, The University of Adelaide, Adelaide, SA, Australia; ^3^National Health and Medical Research Council (NHMRC) Centre of Research Excellence in Digestive Health, University of Newcastle, Newcastle, NSW, Australia; ^4^Hunter Medical Research Institute, New Lambton Heights, NSW, Australia

**Keywords:** disorders of gut-brain interaction (DGBI), functional dyspepsia (FD), functional gastrointestinal disorders (FGID), gastrointestinal (GI), microbiota-gut-brain axis (MGBA), small bowel bacterial overgrowth (SIBO)

Disorders of gut-brain interaction (DGBI), formerly named functional gastrointestinal disorders (FGID), are highly prevalent (Drossman et al., 2016). Although the exact causes are unknown, increasing studies have pointed out a role for gastrointestinal (GI) and central factors through the microbiota-gut-brain axis (MGBA) (Drossman et al., 2016). According to the Rome IV criteria, functional dyspepsia (FD) is characterized by bothersome epigastric symptoms, which are unexplained after routine investigation (Stanghellini et al., 2016). Although there is no evidence for an underlying organic, metabolic or systemic disease process, these criteria do not exclude the presence of microscopic pathology (Talley et al., 2007; Stanghellini et al., 2016). Indeed, significant progress has been made in the search for underlying mechanisms, of which the most important are summarized in the current Research Topic (Figure 1).

**Figure 1:**
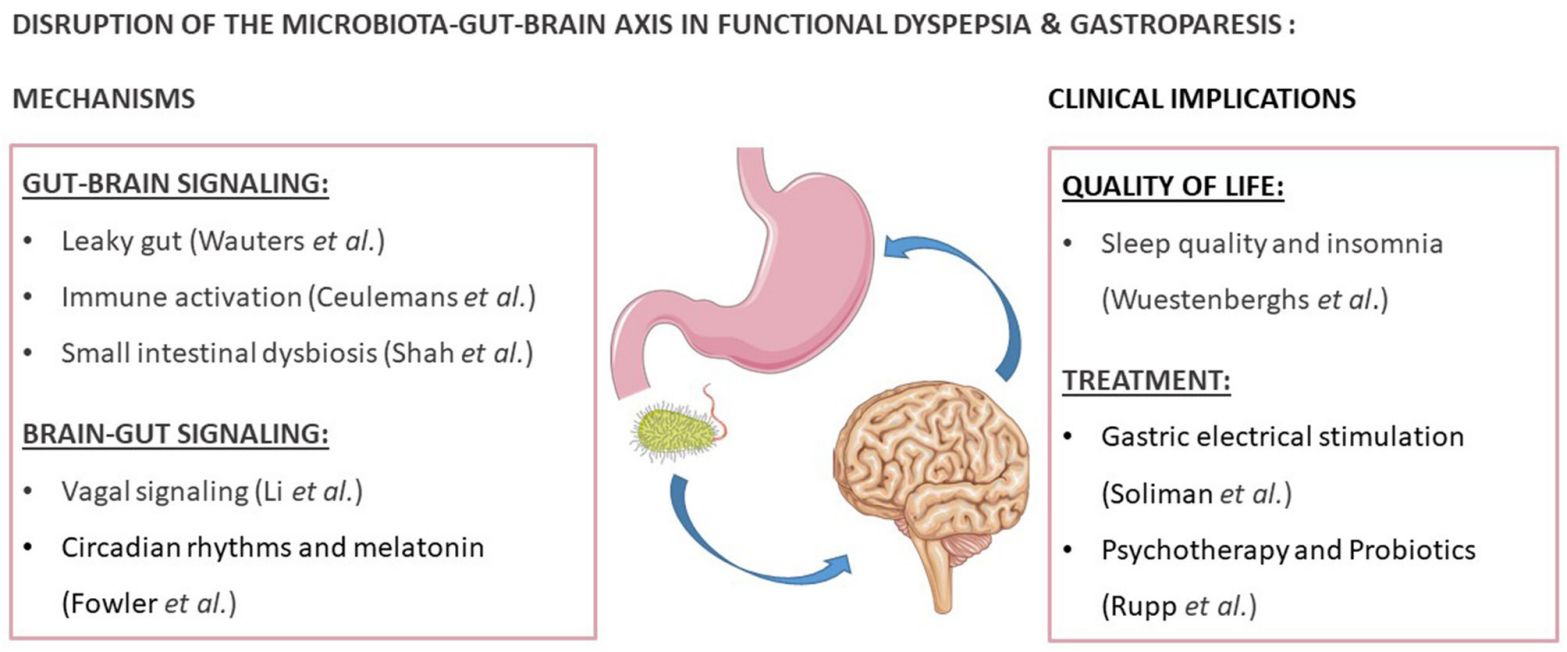
Reviews and research focusing on the disruption of the microbiota-gut-brain axis in functional dyspepsia and gastroparesis. This figure was created with elements from Smartservier.

The proximal small intestine or duodenum has emerged as a key player in GI-disorders as it regulates the passage of food from the stomach to the distal small intestine (Wauters et al., 2020). Changes at the level of the duodenum in FD patients include increased mucosal permeability or “leaky gut,” which could result in an increased influx of antigens into the duodenal mucosa (Wauters et al.). Moreover, immune activation is present at the cellular and molecular level, with increased duodenal eosinophil and mast cell infiltration (Ceulemans et al.). In the reviews in this issue, important methodological aspects, recognized associated factors, and the relevance of increased permeability and inflammation to treatment are discussed (Wauters et al.; Ceulemans et al.). Indeed, both existing therapies such as proton pump inhibitors (PPI) as well as novel barrier-protective or anti-inflammatory drugs may be effective in FD through their effects on improving duodenal permeability and/or inflammation (Ceulemans et al.; Wauters et al., 2021a).

In the duodenum, luminal factors including gastric acid, bile and digestive enzymes limit the microbial population (Wauters et al., 2020). An altered density, diversity and function of the intestinal microbes or “dysbiosis” has been reported in many GI disorders, with the majority of studies in FD focusing on an abnormal load or small intestinal bacterial overgrowth (SIBO) (Gurusamy et al., 2021). Although more challenging to study, novel techniques to study the small intestinal microbiome are emerging as reviewed by Shah et al. Application of these tests will prove useful to study the effects of specific antimicrobial treatments on the duodenal microbiome, as recently shown for first-line PPI-therapy in FD (Shah et al.; Wauters et al., 2021b; Brown et al., 2022).

In addition to duodenal alterations, which may lead to peripheral sensitization and altered neuronal responses or gut-brain signaling (Cirillo et al., 2015), central changes and impaired descending vagal or brain-gut signaling are also present in FD (Li and Page). The different components and potential therapeutic targets of the vagal signaling pathways are reviewed by Li and Page. The vagovagal reflex and its effects on gastric accommodation and emptying are also implicated in gastroparesis, which often presents with nausea and vomiting as well as weight loss in addition to typical functional dyspepsia symptoms (Soliman and Gourcerol; Moshiree et al., 2019). Interestingly, changes in vagal signaling and cerebral activity have been found with gastric electrical stimulation in gastroparesis, which may explain the beneficial effect on nausea and vomiting as reviewed by Soliman and Gourcerol.

Recently, research on circadian rhythms has illustrated its role in regulating GI physiology, including immune responses (Fowler et al.). The potential role of melatonin deserves special attention in this regards, also in relation to the increased fatigue and disordered sleep reported by patients with DGBI as reviewed by Fowler et al. Indeed, the prevalence of altered sleep quality was 81% in FD patients in a study by Wuestenberghs et al. which was associated with lower quality of life, higher depression scores and dyspeptic symptom severity. Therefore, these findings suggest that modulation of the circadian rhythm may be a therapeutic option for FD patients (Fowler et al.; Wuestenberghs et al.). Finally, the efficacy of psychotherapy and probiotics in FD is reviewed by Rupp and Stengel as gut-brain but also brain-gut effects of both treatment approaches have been demonstrated in FD.

In conclusion, the disruption of the MGBA in FD and gastroparesis includes different possible mechanisms, including altered gut-brain and brain-gut signaling. While the duodenal environment seems implicated through several potential pathways in FD, the causality and directionality with symptoms still need to be confirmed. Neuronal alterations are present in both FD and gastroparesis, which may also offer novel therapeutic approaches to treat these frequent but unexplained upper GI disorders. An improved understanding of the MGBA will likely improve both diagnosis and management, ultimately aiming to reduce the significant impact on patients' lives.

## Author Contributions

All authors contributed to the manuscript, revised and approved the final version of the editorial.

## Funding

NT is supported by funding from the National Health and Medical Research Council (NHMRC) to the Centre for Research Excellence in Digestive Health and he holds an NHMRC Investigator grant.

## Conflict of Interest

NT reports, personal fees from, Allakos (gastric eosinophilic disease) (2021), Bayer [IBS] (2020), Planet Innovation (Gas capsule IBS) (2020), Takeda, Japan (gastroparesis) (2019), Viscera Labs, (USA 2021) (IBS-diarrhoea), Dr Falk Pharma (2020) (EoE), Glutagen (2020) (Celiac disease), IsoThrive (2021) (oesophageal microbiome), BluMaiden (2021), Rose Pharma (2021), Intrinsic Medicine (2021), Comvita Mānuka Honey (2021), Reckitt (2022) outside the submitted work. NT has a patent Nepean Dyspepsia Index (NDI) 1998, Biomarkers of IBS licensed, a patent Licensing Questionnaires Talley Bowel Disease Questionnaire licensed to Mayo/Talley, a patent Nestec European Patent licensed, and a patent Singapore Provisional Patent “Microbiota Modulation Of BDNF Tissue Repair Pathway” issued, “Diagnostic marker for functional gastrointestinal disorders” AustraWautersan Provisional Patent Application 2021901692. The remaining authors declare that the research was conducted in the absence of any commercial or financial relationships that could be construed as a potential conflict of interest.

## Publisher's Note

All claims expressed in this article are solely those of the authors and do not necessarily represent those of their affiliated organizations, or those of the publisher, the editors and the reviewers. Any product that may be evaluated in this article, or claim that may be made by its manufacturer, is not guaranteed or endorsed by the publisher.

## Abbreviations

DGBI: disorders of gut-brain interaction
FD: functional dyspepsia
FGID: functional gastrointestinal disorders
GI: gastrointestinal
MGBA: microbiota-gut-brain axis
SIBO: small bowel bacterial overgrowth.
